# Association between selenium intake and migraine: a nationwide cross-sectional study

**DOI:** 10.3389/fnins.2023.1176349

**Published:** 2023-07-18

**Authors:** Leiyong Zhao, Jiahui Yin, Xiaotong Li, Xixue Lu

**Affiliations:** ^1^Department of Psychosomatic Medicine, Affiliated Hospital of Shandong University of Traditional Chinese Medicine, Jinan, China; ^2^College of Traditional Chinese Medicine, Shandong University of Traditional Chinese Medicine, Jinan, China; ^3^Department of Acupuncture, Neck Shoulder and Lumbocrural Pain Hospital of Shandong First Medical University, Shandong First Medical University (Shandong Academy of Medical Sciences), Jinan, China

**Keywords:** association, selenium intake, migraine, non-linear, subgroup analysis

## Abstract

**Background:**

Dietary interventions for migraine are receiving increasing attention. However, it remains unclear whether there is any relationship between migraine and selenium intake. The aim of this study was to investigate the association between selenium intake and migraine.

**Methods:**

We used multivariate logistic regression equations to explore the association between selenium intake and migraine. Restricted cubic splines were used to examine the presence of non-linear relationships. Upon finding a non-linear relationship, a recursive algorithm was used to calculate the inflection point. Population differences were also explored through stratified analysis.

**Results:**

In the model adjusted for all covariates, the ORs (95% CI) for the association between selenium intake and migraine were 0.96 (0.88, 1.04), which was no statistical significance. However, the result of the linear trend test with quadrilles of selenium intake indicated the association between selenium intake and migraine may be non-linear. The restricted cubic splines confirmed this non-linear relationship, finding an inflection point (93.1 mcg/day), where the odds of migraine decreased with increasing selenium intake before the inflection point, and no statistically significant relationship was found after the inflection point. The association between selenium intake and migraine was non-linear in all strata except the obese.

**Conclusion:**

We found a non-linear association between selenium intake and migraine in the general American population.

## Introduction

Migraine is a prevalent neurological disorder characterized by recurrent, mostly unilateral, moderate-to-severe throbbing headaches, often accompanied by nausea, vomiting, photophobia, and phonophobia (Buzzi et al., 2005; Barnett, 2019), and according to the 2016 Global Burden of Diseases (GBD) study, migraine is the second most common neurological disorders (Stovner et al., 2018). The annual prevalence of migraine is 14.4%, with 18.9% in women and 9.8% in men (Ford et al., 2017; Stovner et al., 2018). The annual prevalence of migraine is low in adolescents and older adults, with some studies showing an annual prevalence of about 5% in adolescents and people over 50 years of age, while new-onset migraine-like headaches in people > 50 years require vigilance for secondary headaches (Burch et al., 2019; Ferrari et al., 2022). Migraine has some family aggregation with a heritability rate of 42 (Ducros, 2021).

In the current era, drug research and development have reached a bottleneck, and dietary intervention for chronic diseases is increasingly receiving attention. Modern researches have shown that the antioxidant properties of diet have therapeutic value for neurological diseases (Rizwana et al., 2021; Davis et al., 2022). Flavonoids can reduce cognitive impairment, and relieve depressive moods (Park et al., 2021). Vitamin B12 can inhibit neuronal damage caused by endoplasmic reticulum stress and enhance neural repair and functional recovery after traumatic brain injury (Poole et al., 2022). The intake of the antioxidant zinc is negatively correlated with the incidence of migraine, which indicates that zinc is an important influencing factor for migraine (Liu et al., 2023). The cohort study of Chinese adults showed that dietary selenium was negatively correlated with stroke, indicating that dietary selenium intake should be increased to a certain level to prevent stroke (Shi et al., 2022).

It is well known that selenium is an important antioxidant and an essential trace element for human health, and dietary intake is the main form of body supplementation (Wang et al., 2017; Rayman, 2020). Previous researches have shown that selenium intake can help improve psychoneurological disorders such as stroke, Parkinson’s, and depression through its antioxidant properties (Li et al., 2018; Sun et al., 2022; Zhang et al., 2022). Some migraineurs have lower serum catalase activity, non-oxidative thiol concentrations, and total antioxidant capacity. Meanwhile, the activities of superoxide dismutase in platelets and red blood cells in migraine patients are reduced (Malkki, 2015; Togha et al., 2019). Until now, the association between selenium intake and migraine has never been explored, so we used data from the National Health and Nutrition Examination Survey (NHANES) to assess the association between dietary selenium intake and migraine in order to fill this knowledge gap and provide new evidence for dietary modifications to intervene in migraine.

## Materials and methods

### Study population

**Figure 1:**
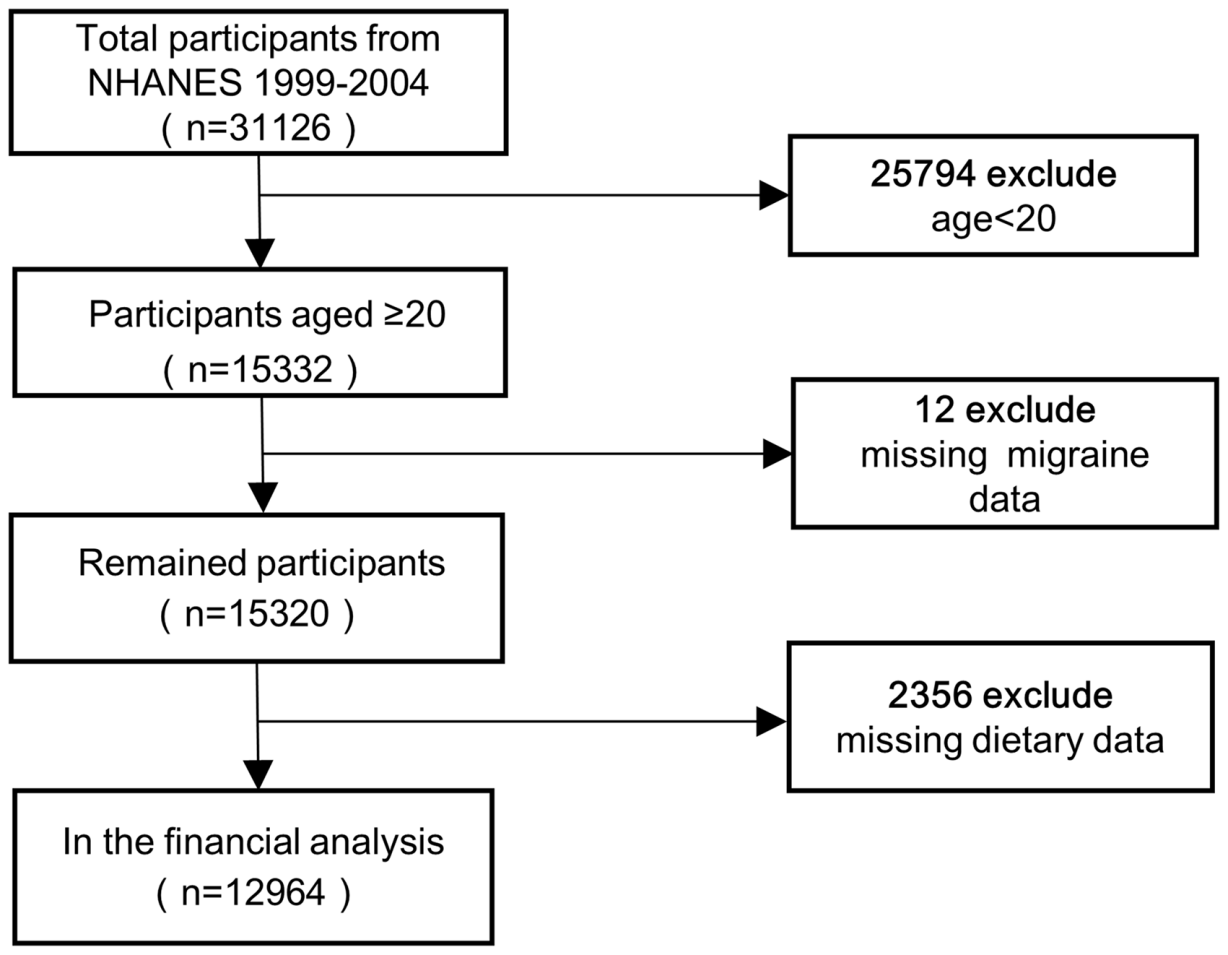
Flow chart of sample selection from the NHANES 1999–2004.

We obtained data from the database for three cycles. The NHANES is a continuously conducted cross-sectional survey that provides self-reported health information on a nationally representative civilian, noninstitutional population of the U.S. All data collection was carried out through home visits, screening by mobile examination centers (MECs), and laboratory testing. The National Center for Health Statistics (NCHS) Ethics Review Committee authorized this cross-sectional study with all participants completing written informed consents. No additional ethical application is required for this study. We combined these data for analysis and obtained a total of 31,126 participants, and our study sample was restricted to adults aged 20 years or older (*N* = 15,332). After excluding participants with missing information on migraine (N = 12) and dietary selenium (N = 2,356), a total of 12,964 study participants remained in our study, of whom 2,645 had migraine (Figure 1).

### Assessment of selenium intake

Data on selenium intake were collected by a 24-h dietary recall interview, conducted by trained dietary interviewers in person. Standard measurement guidelines were provided to participants for reporting food size and quantity. In our study, all participants underwent only the first 24-h dietary recall from 1999 to 2004. We therefore assessed selenium intake based on the first 24-h dietary recall.

### Migraine assessment

The assessment of migraine was based on the question: “During the past 3 months, did you have severe headaches or migraines?.” We were unable to find any other information about migraine in the NHANES. The American migraine prevalence and prevention study indicated that of the 17.4% of participants who reported “severe headache,” 11.8 and 4.6% met the International Classification of Headache Disorders (2nd edition) criteria for migraine and possible migraine, respectively (Buse et al., 2013). Only 1% were classified as “other severe headache.” Thus, we deemed it reasonable to assume that most participants with severe headache experience migraine, the assumption was also made by other researchers in this area (Huang and He, 2022; Li et al., 2022; Peng et al., 2022).

### Covariates

We collected demographic data, lifestyle habits, chronic disease status, body measurements, and biochemical examinations as covariates. Age, sex, race, education level, marital status, and income-to-poverty ratio were incorporated into the demographic variables. Smoking and drinking status were categorized as “current,” “former,” and “never.” The vigorous recreational activities were classified into two categories: yes and no. The medical comorbidities, such as stroke, CVD, hypertension, hyperlipemia, and diabetes, were ascertained by self-reported histories in the questionnaires. The BMI was measured and calculated by trained professionals. Overweight was defined as BMI ≥ 25 kg/m^2^ and < 30 kg/m^2^, and obese was defined with BMI > 30 kg/m^2^. Biochemical examinations include fasting glucose, uric acid, and C-reactive protein.

### Statistical analysis

The study was a secondary analysis of a publicly accessible database. Categorical proportions (%) and means (standard errors, SE) are used for categorical variables and continuous variables, respectively. To compare differences between groups, weighted linear regression models (continuous variables) and weighted chi-square tests (categorical variables) were conducted. Weighted logistic regression analyses were used to explore the relationship between selenium intake and migraine. We used odds ratios (ORs) and 95% confidence interval (95% CI) to report results. Stratified analyses and linear trend-wise tests were also conducted to explore the stability and differences in this relationship across subgroups of the population. We used restricted cubic splines to assess whether there was a non-linear relationship between selenium intake and migraine. A recursive algorithm and a two-segment linear regression model were used to calculate the turning point upon detection of nonlinearity. Statistical analysis was undertaken using R (version 4.2.0) and Empower Stats. Statistical significance was defined as *p* < 0.05.

## Results

### Characteristics of the study population

Table 1 shows the baseline characteristics of the study participants. The mean age of the study population was 46.33 years (95% CI, 45.78, 46.87). Women account for 47.37% of the total 12,964 participants, and 72.03% are white. Compared with people without migraine, migraine sufferers are younger, more likely to be female, have a higher BMI, C-reactive protein, and lower uric acid and income-to-poverty ratio, and are less likely to engage in recreational activities.

**Table 1:** Characteristics of the study population from NHANES 1999–2004.

	Overall (*n* = 12,964)	No migraine (*n* = 10,319)	With migraine (*n* = 2,645)	*p*-Value
Age (years)	46.33 (45.78,46.87)	47.53 (46.86,48.21)	41.96 (41.36,42.56)	<0.0001
Sex (%)				<0.0001
Male	47.37 (46.58,48.15)	51.47 (50.58,52.35)	32.54 (30.41,34.74)	
Female	52.63 (51.85,53.42)	48.53 (47.65,49.42)	67.46 (65.26,69.59)	
Race/ethnicity (%)				<0.0001
Mexican American	7.13 (5.62,8.99)	6.93 (5.47,8.74)	7.84 (6.04,10.12)	
White	72.03 (68.53,75.28)	73.17 (69.82,76.27)	67.90 (63.05,72.39)	
Black	10.43 (8.60,12.60)	10.08 (8.30,12.18)	11.71 (9.39,14.51)	
Other	10.41 (8.05,13.38)	9.82 (7.66,12.51)	12.55 (9.09,17.09)	
Education level (%)				<0.0001
Less than high school	6.97 (6.29,7.71)	6.75 (6.04,7.54)	7.75 (6.71,8.92)	
High school	39.35 (37.41,41.32)	38.06 (35.88,40.30)	43.98 (41.32,46.68)	
More than high school	53.69 (51.53,55.83)	55.19 (52.75,57.60)	48.27 (45.37,51.18)	
Marital status (%)				0.6057
Married/living with partner	64.60 (62.86,66.31)	64.88 (62.98,66.73)	63.62 (61.18,66.00)	
Divorced/separated/widowed	18.74 (17.63,19.91)	18.57 (17.34,19.87)	19.37 (17.55,21.32)	
Never married	16.65 (15.11,18.31)	16.55 (14.96,18.28)	17.01 (14.85,19.40)	
Smoking status (%)				<0.0001
Never	50.39 (48.53,52.24)	50.64 (48.80,52.48)	49.47 (46.40,52.55)	
Former	25.54 (24.13,27.00)	26.97 (25.43,28.56)	20.37 (18.37,22.53)	
Current	24.07 (22.67,25.54)	22.39 (20.89,23.96)	30.15 (27.64,32.80)	
Drinking status (%)				0.0013
Never	12.93 (10.78,15.44)	12.71 (10.50,15.30)	13.73 (11.48,16.34)	
Former	17.75 (16.14,19.47)	16.91 (15.28,18.68)	20.77 (17.88,23.99)	
Current	69.33 (66.10,72.37)	70.38 (67.02,73.53)	65.50 (61.60,69.20)	
Vigorous recreational activities (%)				<0.0001
Yes	33.33 (31.36,35.37)	34.63 (32.41,36.92)	28.63 (26.61,30.75)	
No	66.67 (64.63,68.64)	65.37 (63.08,67.59)	71.37 (69.25,73.39)	
Hypertension (%)				0.0023
Yes	37.87 (36.23,39.53)	38.46 (36.75,40.20)	35.74 (33.66,37.86)	
No	62.13 (60.47,63.77)	61.54 (59.80,63.25)	64.26 (62.14,66.34)	
Hyperlipemia (%)				0.8422
Yes	70.75 (69.47,72.00)	70.69 (69.28,72.06)	70.97 (68.30,73.51)	
No	29.25 (28.00,30.53)	29.31 (27.94,30.72)	29.03 (26.49,31.70)	
CVD (%)				0.9583
Yes	8.92 (8.10,9.81)	8.91 (7.95,9.97)	8.96 (7.56,10.58)	
No	91.08 (90.19,91.90)	91.09 (90.03,92.05)	91.04 (89.42,92.44)	
Stroke (%)				0.0657
Yes	2.56 (2.22,2.95)	2.39 (2.02,2.84)	3.16 (2.46,4.06)	
No	97.44 (97.05,97.78)	97.61 (97.16,97.98)	96.84 (95.94,97.54)	
Diabetes (%)				<0.0001
Yes	12.98 (12.19,13.80)	13.58 (12.72,14.48)	10.77 (9.57,12.11)	
No	87.02 (86.20,87.81)	86.42 (85.52,87.28)	89.23 (87.89,90.43)	
BMI(kg/m2)	28.15 (27.94,28.36)	27.93 (27.71,28.16)	28.92 (28.60,29.24)	<0.0001
Income-to-poverty ratio	2.98 (2.88,3.08)	3.09 (2.98,3.20)	2.60 (2.49,2.71)	<0.0001
Fast glucose (mg/dl)	101.37 (100.39,102.34)	101.75 (100.73,102.78)	99.95 (98.29,101.62)	0.0427
Uric acid (mg/dl)	5.37 (5.34,5.40)	5.45 (5.41,5.49)	5.08 (5.02,5.15)	<0.0001
CPR(mg/dl)	0.43 (0.41,0.45)	0.42 (0.40,0.43)	0.49 (0.45,0.52)	0.0005
Selenium intake(mcg/d)	105.93 (104.52,107.34)	107.62 (106.16,109.08)	99.83 (97.35,102.30)	<0.0001

For continuous variables: survey-weighted mean (95% CI), *p*-value was by survey-weighted linear regression (svyglm). For categorical variables: survey-weighted percentage (95% CI), *p*-value was by survey-weighted Chi-square test (svytable).

CVD, cardiovascular disease; BMI, body mass index; CPR, c-reactive protein.

### Association between selenium intake and migraine

The association between selenium intake and migraine is shown in Table 2. In the model without all variables adjusted, the ORs (95% CI) were 0.90 (0.86, 0.94). However, after adjustment for all covariates, the ORs (95% CI) was 0.96 (0.88, 1.04), which was not statistically significant. Moreover, in the further trend test, the ORs (95% CI) of the association between selenium intake and migraine was Q2 (OR:0.87, 95CI%: 0.69, 1.10), Q3 (OR:0.78, 95CI%: 0.62, 0.99), Q4 (OR:0.81, 95CI%: 0.64, 1.03), and Q5 (OR:0.87, 95CI%: 0.67, 1.11), using Q1 as a reference, indicating that the relationship between selenium intake and migraine might be non-linear. When we performed a sex-stratified analysis, no statistically significant relationship was found. In the age stratification, the study population was not observed to be statistically significant. In the overweight population, the ORs (95% CI) were 0.98 (0.85, 1.12), with the ORs (95% CI) being 0.95 (0.86, 1.05) in the obese population.

**Table 2:** Association between selenium intake and migraine.

	Model 1 OR (95% CI)	*p*-value	Model 2 OR (95% CI)	*p*-value	Model 3 OR (95% CI)	*p*-value
Selenium intake. Z score	0.90 (0.86, 0.94)	<0.0001	0.93 (0.88, 0.97)	0.0118	0.96 (0.88, 1.04)	0.2938
Q1	1.0		1.0		1.0	
Q2	0.84 (0.73, 0.95)	0.0078	0.83 (0.72, 0.95)	0.0180	0.87 (0.69, 1.10)	0.2882
Q3	0.77 (0.68, 0.88)	0.0002	0.77 (0.67, 0.88)	0.0009	0.78 (0.62, 0.99)	0.0402
Q4	0.77 (0.67, 0.88)	<0.0001	0.79 (0.69, 0.91)	0.0100	0.81 (0.64, 1.03)	0.1355
Q5	0.74 (0.64, 0.84)	<0.0001	0.78 (0.67, 0.90)	0.0100	0.87 (0.67, 1.11)	0.2653
*p* for trend	<0.001		<0.001		0.182	
Male	1.03 (0.96, 1.10)	0.4341	0.95 (0.89, 1.02)	0.4916	0.98 (0.87, 1.10)	0.7214
Female	1.00 (0.94, 1.06)	0.9291	0.90 (0.84, 0.96)	0.0079	0.93 (0.83, 1.05)	0.2294
Age						
<60	0.82 (0.78, 0.86)	<0.0001	0.92 (0.87, 0.97)	0.0145	0.96 (0.88, 1.05)	0.3471
≥60	0.88 (0.79, 0.99)	0.0274	0.93 (0.83, 1.05)	0.4601	0.94 (0.76, 1.15)	0.5090
BMI						
Overweight	0.90 (0.85, 0.95)	0.0001	0.92 (0.86, 0.97)	0.0303	0.95 (0.86, 1.05)	0.5609
Obese	0.90 (0.84, 0.97)	0.0066	0.93 (0.86, 1.01)	0.1322	0.98 (0.85, 1.12)	0.2922

Model 1: no covariates were adjusted.

Model 2: age, sex, race were adjusted.

Model 3: Age, sex, race, income-to-poverty ratio, education level, marital status, smoking status, drinking status, hypertension, hyperlipemia, stroke, diabetes, CVD, vigorous recreational activity, BMI, fast glucose, uric acid, and CRP were adjusted.

In the subgroup analysis stratified by age, BMI or sex, the model is not adjusted for the stratification variable itself.

**Figure 2:**
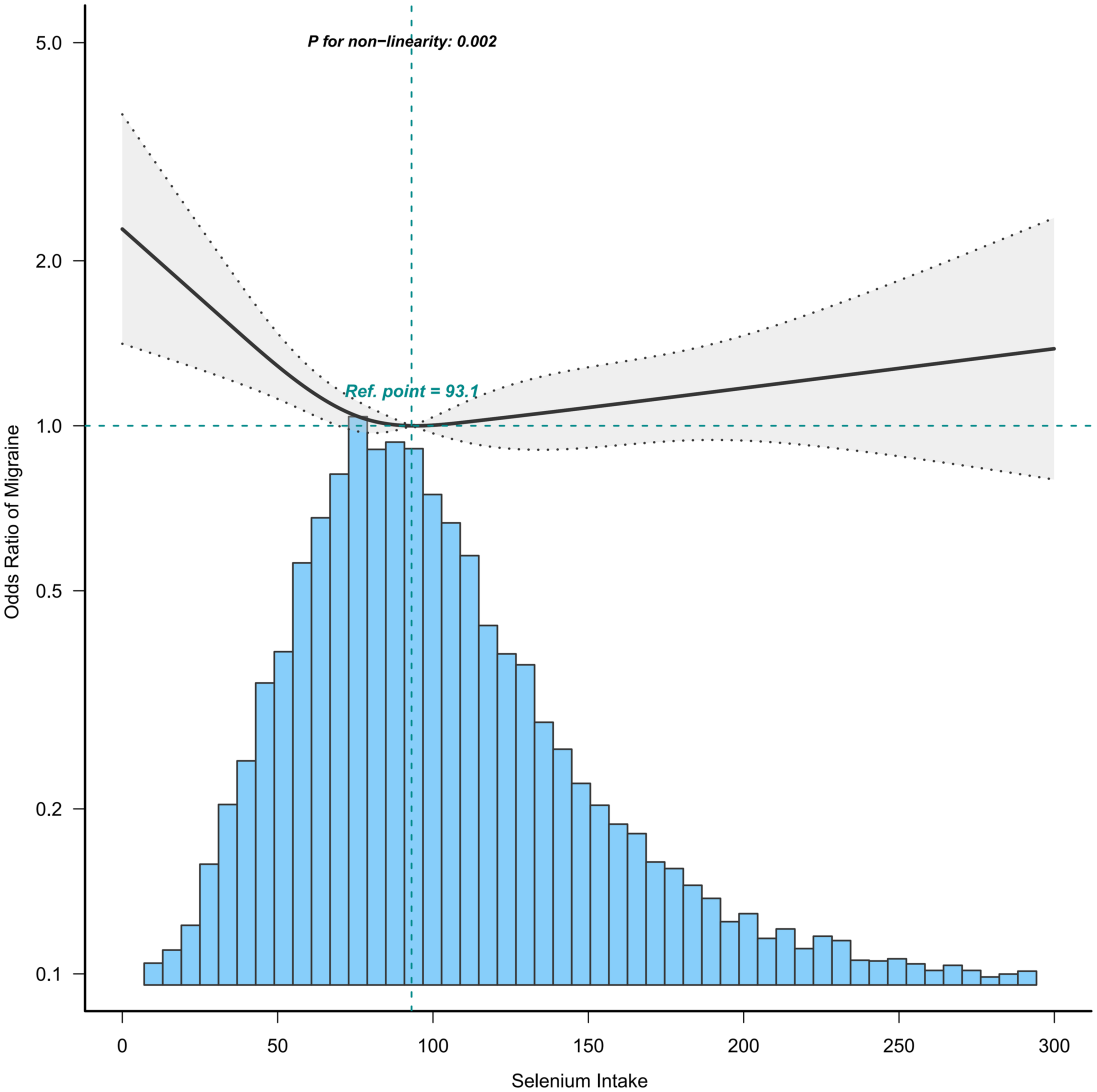
Association between selenium intake and migraine. Age, sex, race, income-to-poverty ratio, education level, marital status, smoking status, drinking status, hypertension, hyperlipemia, stroke, diabetes, CVD, vigorous recreational activity, BMI, fast glucose, uric acid, and CRP were adjusted.

To further explore the relationship between selenium intake and migraine, we used the restricted cubic spline (RCS). When all covariates were adjusted, we confirmed that the association between selenium intake and migraine was non-linear, which may account for the lack of statistical significance (Figure 2). Furthermore, we found the inflection point (93.1 mcg/day), and the relationship before and after the inflection point was reversed, with a negative correlation [0.64(0.49, 0.82)] before the inflection point and a non-statistically significant positive correlation [1.04 (0.90, 1.20)] after the inflection point (Table 3).

**Table 3:** Threshold effect analysis of selenium intake on migraine using a two-piecewise linear regression model.

Subgroups	Inflection point	OR (95%CI)	*p*-value
Total			
	<93.1	0.64 (0.49,0.82)	0.0006
	>93.1	1.04 (0.90,1.20)	0.6187
Sex			
Men			
	<108.2	0.83 (0.66,1.04)	0.1109
	>108.2	1.37 (1.01,1.93)	0.0462
Women			
	<82.25	0.27 (0.11,0.66)	0.0042
	>82.25	1.06 (0.91,1.24)	0.4833
Age			
<60			
	<99.66	0.77 (0.61,0.97)	0.0285
	>99.66	1.06 (0.88,1.27)	0.5663
≥60			
	<83.25	0.37 (0.17,0.82)	0.0138
	>83.25	1.18 (0.85,1.62)	0.3118
Overweight			
	<92.7	0.39 (0.21,0.73)	0.0033
	>92.7	1.08 (0.95,1.23)	0.257

Age, sex, race, income-to-poverty ratio, education level, marital status, smoking status, drinking status, hypertension, hyperlipemia, stroke, diabetes, CVD, vigorous recreational activity, BMI, fast glucose, uric acid, and CRP were adjusted. In the subgroup analysis stratified by age, BMI or sex, the model is not adjusted for the stratification variable itself.

**Figure 3:**
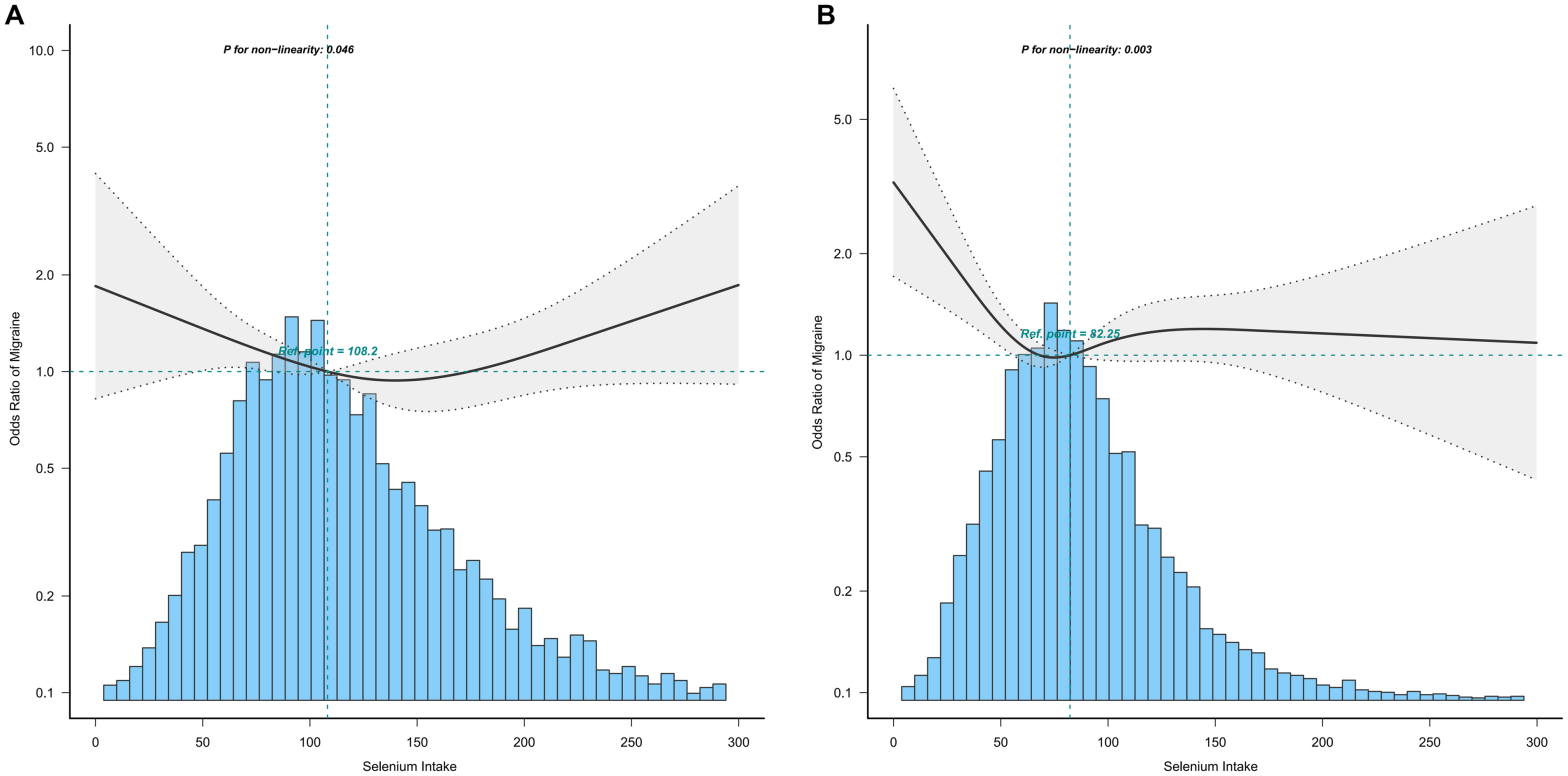
Association between selenium intake and migraine stratified by sex (**A**: men; **B**: women). In the subgroup analysis stratified by sex, the model is not adjusted for sex.

**Figure 4:**
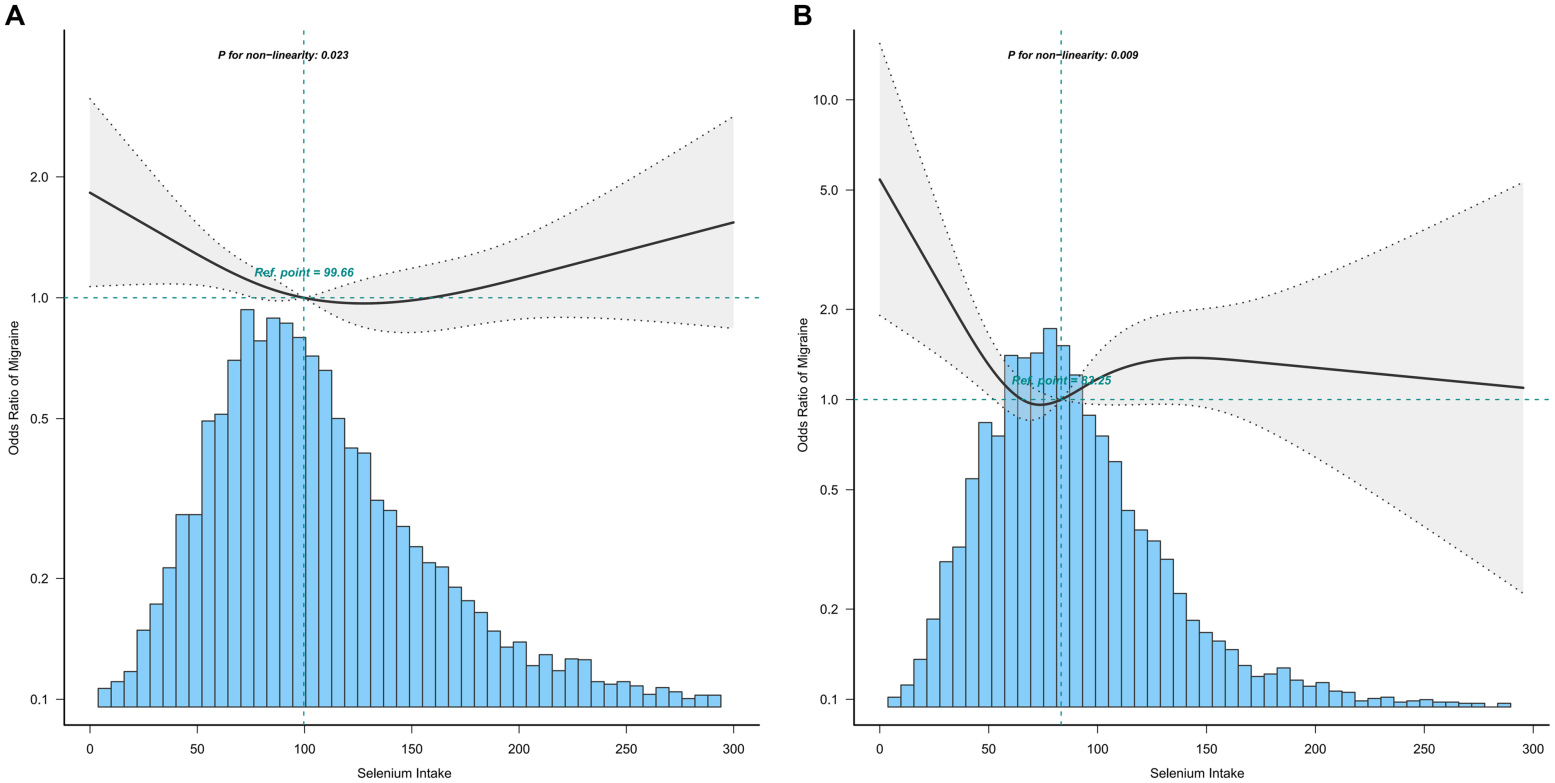
Association between selenium intake and migraine stratified by age (**A**: age < 60; **B**: age ≥ 60). In the subgroup analysis stratified by age, the model is not adjusted for age.

**Figure 5:**
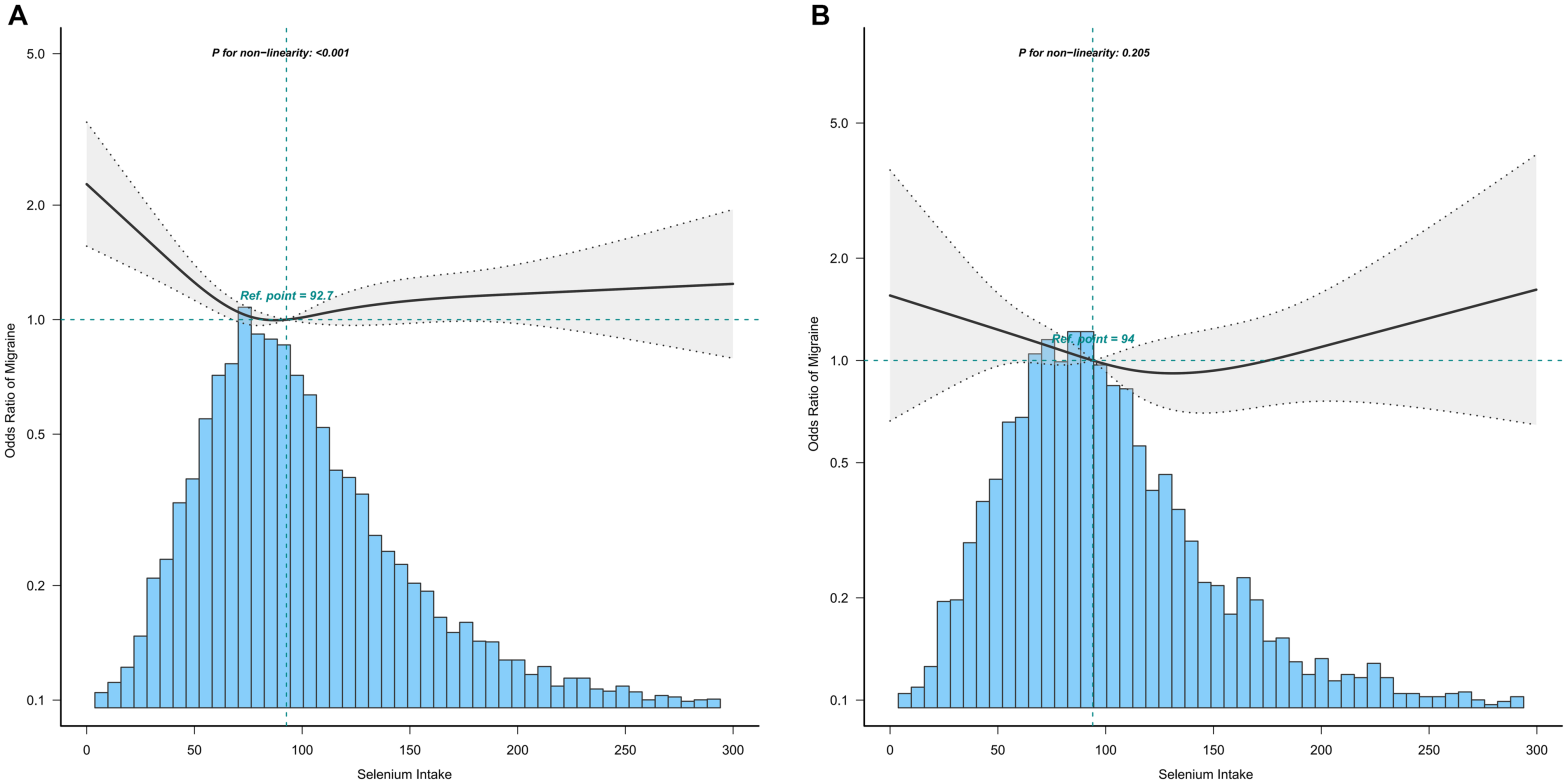
Association between selenium intake and migraine stratified by BMI (**A**: overweight; **B**: obese). In the subgroup analysis stratified by BMI, the model is not adjusted for BMI.

The association between selenium intake and migraine was found to be non-linear in all subgroups of the population except the obese when the stratification was performed (Figures 3–5). In the sex stratification, the inflection point for men was 108.2 mcg/day, and the relationship was only statistically significant at 1.37 (1.01, 1.93) after the inflection point (Table 3). However, the negative association in women was observed only before the inflection point (82.25mcg/day; Table 3). The risk of depression decreased by 23% with increasing selenium intake in people aged < 60 only when selenium intake was less than 99.66mcg/day (Table 3). In the elderly population, the inflection point was 83.25 mcg/day (Table 3). In overweight adults, the relationship was statistically significant only before the inflection points [0.39 (0.210, 0.73)] (Table 3). There was no non-linear relationship in the obese population (*P* for non-linearity = 0.206; Figure 5B).

## Discussion

In this exploration with a United States population, no significant association between selenium intake and migraine was found after adjusting for all covariates. In a further restricted cubic spline, we observed a non-linear relationship between selenium intake and migraine, with an inflection point of 93.1mcg/day, where the odds of migraine decreased with increasing niacin intake before the inflection point, and no statistically significant relationship was found after the inflection point. The association between selenium intake and migraine was non-linear in all strata except the obese. To our knowledge, this is the first study to explore the association between selenium intake and migraine and perform stratified analyses to explore differences among populations in subgroups.

The association between the antioxidant selenium and migraine is rarely studied. A case–control study indicated migraine patients had significantly lower selenium levels compared to healthy participants. Individuals in the lowest quartile of selenium levels were approximately 11 times more likely to have migraine than those in the highest quartile of selenium levels (Talaie et al., 2022). Animal experiments have shown that selenium supplementation improves migraine in model rats by modulating oxidative stress and antioxidant redox systems in the brain, with protective effects on MMCA, GSH, GSH-Px, and antioxidant vitamin concentrations (Naziroglu et al., 2015).

Stratified analysis was performed to further estimate the association between selenium intake and migraine in different groups. In the stratified analysis before performing restricted cubic splines, we did not find a significant relationship between selenium intake and migraine. Upon the restricted cubic splines, the relationships between selenium intake and migraine were non-linear in all subgroups of the population except the obese, and a clear relationship existed only before the inflection point in all subgroups of the population, except men and the obese. In men, the relationship was statistically significant both before and after the inflection point.

Selenium is an essential immune nutrient with anti-inflammatory, antioxidant, and immunomodulatory effects (Huang et al., 2012). However, from the toxicological viewpoint, selenium becomes extremely toxic when its concentration is slightly above its nutritional level (Huang et al., 2022). Selenium (especially organic selenium) provides important regulatory effects on neuromodulatory systems such as the cholinergic and glutamatergic systems, as well as enzymes such as delta-ALA-D and acetylcholinesterase (Dominiak et al., 2016; Naziroglu et al., 2020). On the other hand, the interaction of selenium derivatives with metal-induced toxicity has produced conflicting results. Although ameliorative and neutral effects of organic and inorganic selenium on non-essential metal toxicity are frequently reported, substantial evidence demonstrates the potentiating effect of selenium on the neurotoxicity of non-essential metals such as mercury, cadmium, and lead, which reduces the antioxidant capacity of neuronal cells, while increasing their susceptibility to further oxidative damage (Nogueira and Rocha, 2011; Lv et al., 2021; Naderi et al., 2021).

Selenium intake in certain amounts can help alleviate migraines, and the probable mechanism may be related to inflammation and oxidative stress. Migraine is recognized to be associated with increased mitochondrial energy metabolism (Cevoli et al., 2019). Mitochondrial oxidative stress is important in the pathophysiology of migraine, and selenium has a regulatory effect on mitochondrial oxidative stress in the brain (Fila et al., 2022). Oxidative stress is controlled by antioxidants, including selenium. Selenium plays an important role in the nervous system, including the brain, as a cofactor for glutathione peroxidase and is incorporated into selenoproteins involved in antioxidant defense. It is neuroprotective by modulating excessive ROS production, inflammation, and Ca2 + overload in several diseases, including inflammatory pain, allergic, atopic pain, diabetic neuropathic pain, and injurious pain (Steinbrenner and Sies, 2013; Hariharan and Dharmaraj, 2020).

The occurrence of migraine can be exacerbated when selenium is consumed in excess, although with no statistical significance. There is substantial evidence that excessive selenium intake may disrupt the normal function of various key proteins and signaling molecules engaged in oxidative stress and neuroinflammation, leading to an increased inflammatory response (Benton, 2002; Rayman et al., 2018; Zhao et al., 2022). Elevated selenium exposure is also suspected to be associated with neuropsychiatric disorders such as stroke, depression, and headaches (Colangelo et al., 2014; Hu et al., 2017).

Our research has some advantages. First, we adopted a large, nationally representative, and rigorously quality-controlled dataset, which increases the reliability of our results. Second, such a large sample size enables us to conduct further subgroup analysis, exploring the differences in the relationship between selenium intake and migraine among different populations, which is beneficial for formulating different intervention measures according to specific populations. Third, with the adjustment of potential confounders, our conclusions are more in line with reality. However, this study also has several limitations. First, as a cross-sectional study, due to the nature of observational studies, we cannot exclude reverse causality, nor can we reveal the causal relationship between the dependent and independent variables. Second, dietary data were collected from a 24-h dietary recall, so they cannot be used to represent long-term dietary intake, and it is not possible to determine whether the relationship between selenium intake and migraine will change over time. Third, the use of 24-h dietary recall depends on memory, so it is prone to over-reporting or under-reporting, and may not reflect habitual intake. Fourth, other potential confounders not adjusted in this study may still cause bias.

## Conclusion

Using the NHANES database study, a non-linear association between selenium intake and migraine was found with subgroup differences being explored. Our study may help clinicians and public health departments to better design relevant programs to help prevent and treat migraine. More basic experiments need to be conducted to explore the mechanisms by which selenium acts in migraine.

## Data availability statement

The original contributions presented in the study are included in the article/supplementary material, further inquiries can be directed to the corresponding author.

## Ethics statement

The studies involving human participants were reviewed and approved by National Center for Health Statistics (NCHS) Ethics Review Committee. The patients/participants provided their written informed consent to participate in this study.

## Author contributions

LZ and JY: project development and research design and manuscript writing. XLu: responsibility for data analysis and interpretation, manuscript writing. XLi: manuscript editing and interpretation of the results. All authors contributed to the article and approved the submitted version.

## Funding

This work was supported by Shandong Province Chinese Medicine Science and Technology Project (grant number 2021M150).

## Conflict of interest

The authors declare that the research was conducted in the absence of any commercial or financial relationships that could be construed as a potential conflict of interest.

## Publisher’s note

All claims expressed in this article are solely those of the authors and do not necessarily represent those of their affiliated organizations, or those of the publisher, the editors and the reviewers. Any product that may be evaluated in this article, or claim that may be made by its manufacturer, is not guaranteed or endorsed by the publisher.
